# Supporting Female Survivors of Gender-Based Violence Experiencing Homelessness: Outcomes of a Health Promotion Psychoeducation Group Intervention

**DOI:** 10.3389/fpsyt.2020.601540

**Published:** 2020-12-09

**Authors:** Ali Bani-Fatemi, Monica Malta, Amanda Noble, Wei Wang, Thanara Rajakulendran, Deborah Kahan, Vicky Stergiopoulos

**Affiliations:** ^1^Centre for Addiction and Mental Health, Toronto, ON, Canada; ^2^Covenant House, Toronto, ON, Canada; ^3^Department of Psychiatry, University of Toronto, Toronto, ON, Canada

**Keywords:** quality of life, homelessness, psychoeducation, mixed methods, trauma-informed intervention, gender-based violence (GBV), victimization

## Abstract

Homelessness is an important risk factor for gender-based violence (GBV), particularly among youth, and disproportionally affects women and girls. Survivors of GBV experience enduring and severe physical, psychological, and sexual health problems. Although key elements in service delivery for survivors of GBV have been identified, little is known about outcomes of community-based programs aiming to assist homeless and unstably housed youth experiencing GBV. This longitudinal study aimed to quantitatively evaluate changes in mental health and well-being outcomes in female identified youth experiencing GBV and homelessness, 12 months after enrolment in a community-based, trauma-informed, brief group psychoeducation intervention. Standardized survey measures were administered at baseline, 6 and 12 months for 70 participants, recruited between February 2017 and April 2019, assessing quality of life, psychological distress, traumatic symptoms, substance use, resilience, victimization, and sense of mastery. Linear mixed models were used to examine longitudinal changes in quality of life as well as secondary outcomes among study participants. After 12 months, quality of life increased significantly among participants (*p* = 0.009), and the 12-month victimization score was significantly decreased relative to baseline (*p* = 0.05). Changes in other outcomes were not statistically significant. Findings suggest that community-based brief group psychoeducation interventions may be a promising approach to improving outcomes for this disadvantaged population.

## Introduction

Gender-based violence (GBV) defined as any harmful act committed against a person because of his/her gender (1, 2) and disproportionately affects women and girls (3). Globally, one out of three women reports having been a victim of gender-based physical or sexual violence (4), including physical violence, sexual violence (such as sex trafficking, sexual exploitation, and sexual assault), psychological or emotional violence, and economic violence. Studies have shown that homelessness in youth is an important risk factor for GBV, and homeless youth are more vulnerable to sexual exploitation and trafficking (5–7). Furthermore, the proportion of homeless women who have experienced GBV may be underestimated, as women are believed to be overrepresented among the hidden homeless, and as a result less likely to appear in homelessness data (8).

The impact of GBV is experienced long after the violence ends. The health-related impact of GBV includes physical injuries (9), chronic pain syndromes, memory problems, urogenital, cardiovascular, musculoskeletal, dermatological, eye, ear, and upper respiratory complaints, forced abortions, cervical cancer, and sexually transmitted infections (10–12). Mental health problems include depression, anxiety, posttraumatic stress disorder (PTSD), anger, dissociative disorders, grief, guilt, aggressive behaviors, self-harm and suicidal ideation (13–16), as well as substance use and dependence (17).

Apart from life-threatening physical and mental health consequences, survivors of GBV face a number of social consequences, including social isolation and marginalization (18). Lack of trust in the police, social services, or those in positions of authority may further isolate victims from seeking support or assistance (19).

Given the enduring sequelae of GBV, at risk populations, such as youth experiencing homelessness, have a high need for social and community supports. Recent literature reviews of strategies to prevent or reduce GBV or its sequelae suggest that although a number of interventions have shown promising findings, the paucity of evaluative efforts has led to inconsistencies in intervention activities and characteristics across settings (20). Interventions for intimate partner violence are extensively studied and offer women-centered, psychosocially supportive approaches that may be efficient in primary and secondary prevention of intimate partner violence (21). Moreover, trauma informed group therapy has been beneficial in reducing the negative psychological impacts experienced by survivors of sexual assault (22, 23), leading to a reduction in post-traumatic stress symptoms and risk-taking behaviors, and improvements in perceived empowerment, knowledge, and application of coping strategies (22). Although a wide range of interventions to support youth experiencing homelessness have been identified, including individual and family therapies, skill building programs, case management, and structural interventions (24), little is known about community programs aiming to assist youth experiencing GBV and homelessness or housing instability. The dearth of research in this area leaves service providers (e.g., counselors, social workers, and therapists) with little guidance on how to best support this population.

This longitudinal observational study, part of a realist informed evaluation using mixed methods, aimed to quantitatively evaluate changes in quality of life (primary outcome), psychological distress, traumatic symptoms, substance use, resilience, victimization, and sense of mastery (secondary outcomes) in young women experiencing GBV and homelessness, 12 months after enrolment in a community-based, trauma-informed, group psychoeducation intervention. It was hypothesized that the intervention may lead to improved quality of life, resiliency, and a sense of mastery and reduced mental health symptoms, substance use and victimization in this population.

## Methods

### Setting

Covenant House Toronto (CHT) is a large community-based organization in Toronto, Canada, offering an on-site crisis shelter, school, and transitional housing services, as well as counseling, health care, employment assistance, and other community-based services to youth experiencing homelessness. Given the high prevalence of GBV in this population, CHT, funded by the Public Health Agency of Canada, launched as part of a broader 5-year strategy to support women and girls experiencing GBV and homelessness, a brief group psycho-education health promotion intervention, the Peer Education and Connection through Empowerment (PEACE) program.

### Intervention Description

The PEACE program, launched in 2017, is a community-based group psychoeducation intervention for female identified youth aged 16 to 24 experiencing GBV and homelessness (25). Co-facilitated by trained peer mentors, the program has sought to empower and support survivors of GBV, offering weekly trauma-informed psychoeducation groups over 16 weeks to promote health and well-being. The program has used a community development participatory action framework wherein program participants become active agents of change to address power differentials, intersectional issues, systemic barriers to care, and well-being and social isolation—all of which are major concerns for this vulnerable population. Under the mentorship of the health promotion coordinator and peer mentors, participants are supported and encouraged to engage in discussions and share ideas for the curriculum based on their needs and preferences. Content typically includes identity formation, self-image, women's health, healthy relationships, coping mechanisms, and leadership. Moreover, participants engage in a range of social activities, including yoga, arts and crafts, and meal preparation. Groups have approximately 8 participants each in order to allow for a safe and confidential space as well as a high level of support during the intervention. A mental health counselor and a mobile crisis team are also available to support participants and connect to other resources and supports as needed. To evaluate the intervention, CHT partnered with the Center for Addiction and Mental Health (CAMH), affiliated with the University of Toronto. The study protocol was approved by both the CAMH and the University of Toronto Institutional Research Ethics Boards.

### Study Design and Participants

The present study was part of a non-experimental realist-informed longitudinal evaluation using mixed methods to enable a rich understanding of the program and its context and explore ideas and outcomes relevant to the needs of knowledge users (26, 27).

Qualitative methods were used to examine barriers to implementation (28), as well as participant perspectives on the impact of the intervention on their wellness and recovery (29). In these studies, participants described valuing the safe, respectful, women-only space, the multiple shared lived experiences, and tailored psychoeducation, and reported perceived improvements in self-confidence, coping ability, health, relationships, and future directedness, as a result of participation (29). Notably, there were no premature drop-outs from the program during the study period.

**Figure 1:**
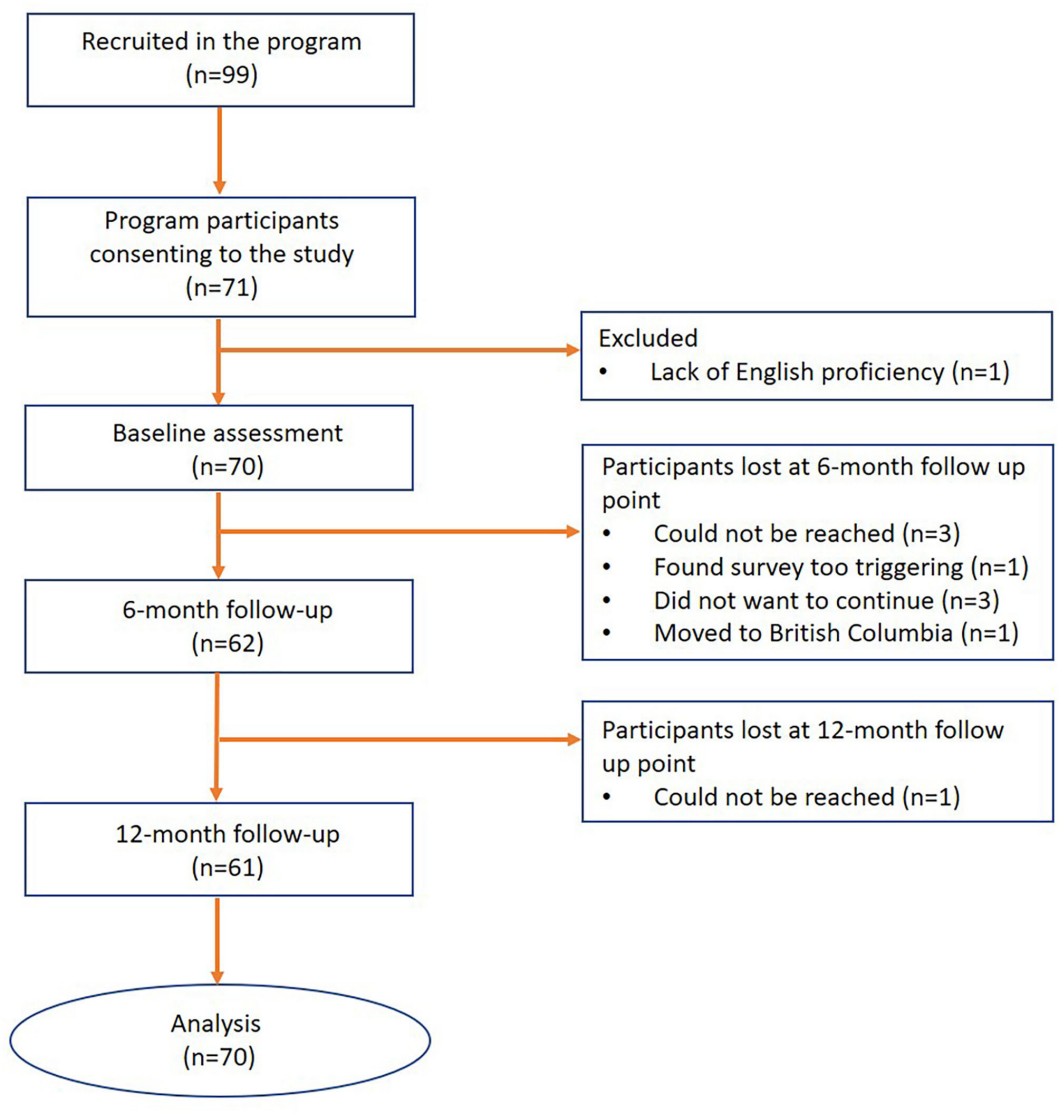
Study flowchart.

The present study describes key participant outcomes 12 months after program enrolment. Study participants (*n* = 70) were recruited among successive service users of the PEACE program between February 2017 and April 2019 (Figure 1). The study drop-out rate at 6- and 12-months of follow-up were 11.43 and 1.78%, respectively (Figure 1). Written informed consent was obtained from all participants. Inclusion criteria for the study included: survivors of GBV aged between 16 to 24 years, enrollment in the PEACE program, verbal proficiency in English, and capacity to consent to research participation.

### Data Collection

Several standardized survey measures, detailed below, were administered by trained research staff at baseline, 6-month, and 12-months. The primary outcome examined was quality of life. Secondary outcomes included victimization, resiliency, psychological distress, substance use, level of mastery, and traumatic stress symptoms among study participants. Demographic information was collected at baseline using survey questions developed for the study by the principal investigator.

#### Survey Measures

##### WHO Quality of Life, Shortened Version (WHOQOL-BREF)

This standardized measure, including 26 questions, assesses participants' quality of life in four domains—(2) Physical health, (3) Psychological health, (5) Social relationships, and (6) Environment. Using the measure, it is possible to drive four domain scores, in addition to a total score. Domain scores are scaled in a positive direction that means higher scores denote a higher quality of life. WHO QOL-BREF domain scores have demonstrated good discriminant validity, content validity, internal consistency and test & retest reliability. The measure was also found to have good to excellent reliability and validity (30).

##### Hospital Anxiety and Depression Scale (HADS)

This standardized measure is a self-report rating scale of 14 items on a 4-poing Likert scale ranging between 0 and 3. The HADS produces two subscales: the A-scale measuring Anxiety and the D-scale measuring Depression, which have 7 items each and are scored separately. The A-scale covers the state of anxious mood, restlessness, and anxious thoughts. The D-scale focuses upon loss of interest and diminished pleasure response. The scale is validated among adolescents (31) as well as adults (32). Scores range from 0 to 21 for each subscale with higher scores suggesting higher severity of anxiety or depression.

##### Connor-Davidson Resiliency Scale (CD-RISC2)

The CD-RISC2 is a two-item scale that is a briefer version of the full 25-item CD-RISC (33) and was developed as a measure of bounce-back and adaptability. The CD-RISC2 has demonstrated significant correlation with both the overall CD-RISC score and with the individual items of the CD-RISC, suggesting that the two items of the CD-RISC2 are appropriate representatives of the overall scale and the CD-RISC2 can be utilized in place of the CD-RISC (33). Similar to CD-RISC, the 2-item scale shows good test-retest reliability, convergent validity, and divergent validity. The CD-RISC2 range from 0 to 8 and higher scores indicate higher resilience.

##### Global Assessment of Individual Need—Substance Problem Scale (GAIN-SPS)

To measure substance use severity, we used the 7-item GAIN-SPS scale (34). The severity of substance use problems is calculated by summing the number of substance-related problems reported in the past month (range from 0 to 5). The GAIN-SPS has been found to have excellent internal consistency, good concurrent validity with objective measures of substance use, and good discriminant validity for detecting the presence of a substance use diagnosis (34). A higher score shows higher substance use severity.

##### Adverse Childhood Experiences (ACE)

This standardized measure captures the extent and nature of childhood experiences of neglect and abuse as well as family dysfunction such as domestic violence and alcohol or drug problems. The ACE is a 10-item self-report measure and has been found to have good reliability among adolescents (35) as well as adults (36). Each positive answer is assigned one point and the final score (0–10) is known as the ACE score. Participants with more difficulties in childhood have a higher ACE score.

##### Selected Questions From 2014 Stats Canada General Social Survey (GSS) Victimization Module

Selected questions focusing on physical violence and other kinds of crime was used to assess participants' experiences of victimization within the past six months. The GSS has been extensively used in Canadian populations aged 15 and over (37). Higher total scores indicate higher experience of victimization.

##### Pearlin Mastery Scale

This standardized measure by Pearlin and Schooler (38) was used to assess how strongly participants agreed with statements asserting feelings of self-mastery, an aspect of coping skills. This 7-item scale includes five negatively worded items and two positively worded items on a 4-point Likert scale ranging between 1 and 4. The negatively worded items need reverse coding prior to scoring, resulting in a score range between 7 and 28. Higher scores represent greater levels of mastery. The psychometric properties are well tested (38) and several studies have tested its validity among homeless adult populations (39).

##### UCLA-PTSD Reaction Index—DSM V (excluding clinical assessment section)

This scale was used to assess presence or absence of posttraumatic stress symptoms among study participants. The earlier version of the scale (UCLA-PTSD Reaction Index—DSM IV) has been used to assess a variety of traumatic experiences such as natural disasters, political violence, school/community violence (40). The scale assesses the frequency of occurrence of PTSD symptoms during the past month rated from 0 (none of the time) to 4 (most of the time). Participants with higher traumatic stress symptoms have higher score. The scale has been found to have good reliability and validity for adults as well as youth between 16 to 18 years of age (41).

### Data Analysis

To address missing data pertaining to loss to follow up, missing scales, or missing items within scales, multiple imputation by chained equations (MICE) was conducted using the MICE package in R (42). The dataset with all variables, including baseline and each follow-up point was used, assuming missing at random (MAR). Further analyses were carried out using SPSS Statistics, version 25. Descriptive statistical analysis (means and standard deviation) was performed to examine participants' socio-demographic characteristics, mental health, alcohol and substance use, and quality of life. Linear mixed models were used to examine longitudinal changes in outcomes among study participants. We evaluated the effect of time (in months), baseline covariates, and the interaction between time and covariates. To avoid overfitting by including too many covariates, we only included variables for which there was a significant bi-variate association with quality of life including ACE, race, and place of birth (*p* < 0.05).

## Results

### Demographic and Clinical Characteristics

Table 1 shows the baseline socio-demographic characteristics and ACE scores of study participants. The mean age of participants at enrollment was 21.47 years (SD: 3.79). Nearly half of the participants (48.6%) were born in Canada. Over a third of participants were black (37.2%), while 22.8% were white and 40% reported other ethnicities; Two individuals (2.8%) reported being married, while 11 participants (16%) reported having children. Two of the participants (2.8%) identified as transgendered, one participant disclosed her gender as both female and male, and one participant did not disclose their gender.

**Table 1:** Demographic characteristics of study participants (*N* = 70).

**Demographic variables**		**Frequency (*n*)**	**Percent (%)**
Race	Black	26	37.1
	White	16	22.9
	Other	28	40
Education Level	Completed grade 5-8	2	2.9
	Attended high school (not completed)	30	42.9
	Completed high school	17	24.3
	Attended business, trade, technical school	7	10
	Attended University, not completed	8	11.4
	Completed undergraduate education	6	8.6
Participants with Children	-	11	15.7
Continuous Work in the Past Year	-	9	12.9
Number of Adverse Childhood Experiences (ACE score)	0	3	4.3
	1	3	4.3
	2	1	1.4
	3	5	7.1
	4 or more	58	82.9

Nearly half (45.7%) of the participants did not complete high school, 24.3% completed high school, and 30% had some postsecondary education. The average total ACE score among participants was 6.14 (SD: 2.90), with most participants (82.9%) having a total ACE score of 4 or more, and only 4.3% reporting no adverse childhood experiences. The most common ACE reported was emotional abuse (75.7%), while physical neglect was the least commonly reported ACE (34.3%). The most common type of GBV described was family violence, reported by 64% of the participants. Many of our participants (*n* = 45 or 64.3%) experienced more than one type of GBV, while three of the participants (4.3%) did not disclose the type of violence experienced. To identify what baseline variables were associated with the outcomes of interest, a linear regression analysis of demographic variables in association with the outcome measures at baseline was conducted. ACE scores were found to be negatively associated with psychological health (*t* = −2.33, *p* = 0.02) and overall quality of life (*t* = −2.99, *p* = 0.004) and positively with victimization (*t* = 2.23, *p* = 0.029), while race was negatively associated with physical health (*t* = −2.06, *p* = 0.04), psychological health (*t* = −2.19, *p* = 0.03), and overall quality of life (*t* = −2.83, *p* = 0.006). After adjusting for race, the total ACE score was significantly associated only with overall quality of life (AOR, 95% CI: 0.70, 0.54-0.91). In addition, victimization (1.65, 0.97-2.79), psychological health (0.83, 0.67-1.02), and traumatic stress symptoms (1.02, 0.99-1.05) showed a trend toward a higher total ACE score.

At baseline participants born in Canada had a significantly lower score of physical health (1.41, 1.13-1.76), psychological health (1.25, 1.06-1.47), environment (1.41, 1.09-1.83), overall quality of life (1.28, 1.07 = 1.52), and a significantly higher score of HADS-anxiety (0.80, 0.69 = 0.93), and traumatic stress symptoms (0.97, 0.95-0.99) compared to those who migrated to Canada. In addition, immigrants to Canada had significantly higher substance use problem scores (0.26, 0.08-0.85) than individuals with temporary status in Canada (work permit, domestic help, visitor, student, refugee claimant).There were no significant differences in baseline outcome measures between participants with and without children, complete and incomplete high school, and participants with and without continuous work in the past year.

### Primary and Secondary Outcomes at 12 Months

The overall quality of life score increased significantly over 12 months (*F* = 5.585, *p* = 0.004) (Table 2) among participants, although increases in individual domains of quality of life were not significant.

**Table 2:** Outcomes of a brief psychoeducation group intervention for female identified youth survivors of GBV experiencing homelessness (*N* = 70).

**Outcomes**	**Baseline**	**6-month**			**12-month**			**Overall *p*-value**
	**Mean (SD)**	**Mean (SD)**	**Difference from baseline (95% CI)**	***p*-value**	**Mean (SD)**	**Difference from baseline (95% CI)**	***p*-value**	
WHOQOL (Physical health)	13.96 (2.71)	14.45 (2.54)	−0.488 (-1.650 to 0.674)	0.93	14.49 (2.69)	−0.527 (-1.702 to 0.648)	0.84	0.46
WHOQOL (Psychological health)	12.36 (3.47)	13.39 (3.43)	−1.032 (-2.512 to 0.447)	0.28	13.37 (3.19)	−1.010 (-2.505 to 0.486)	0.31	0.15
WHOQOL (Social relationship)	13.28 (3.30)	13.63 (3.79)	−0.353 (-1.903 to 1.196)	0.99	13.76 (3.57)	−0.480 (-2.047 to 1.086)	0.99	0.74
WHOQOL (Environment)	13.15 (2.37)	13.89 (2.60)	−0.738 (-1.810 to 0.334)	0.29	13.90 (2.39)	−0.753 (-1.836 to 0.331)	0.28	0.14
WHOQOL (Overall)	12.54 (3.43)	14.11 (3.26)	−1.568 (-3.028 to−0.108	**0.03**	14.38 (3.27)	−1.842 (-3.317 to−0.366)	**0.009**	**0.004**
Victimization	1.63 (1.86)	0.91 (1.44)	0.721 (-0.014 to 1.457)	0.057	0.88 (1.64)	0.744 (0.0004 to 1.487)	**0.050**	**0.02**
HADS – A Scale	10.86 (4.00)	9.65 (4.63)	1.209 (-0.635 to 3.053)	0.34	10.06 (4.02)	0.799 (-1.064 to 2.663)	0.90	0.27
HADS – D Scale	7.04 (3.95)	6.24 (3.10)	0.802 (-0.778 to 2.383)	0.66	5.96 (3.62)	1.158 (-0.439 to 2.756)	0.24	0.19
GAIN-SPS	0.77 (1.34)	0.54 (0.92)	0.234 (-0.284 to 0.753)	0.83	0.54 (1.19)	0.233 (-0.291 to 0.757)	0.85	0.44
UCLA-PTSD	58.84 (25.99)	49.61 (26.38)	9.232 (-2.455 to 20.919)	0.17	51.63 (27.91)	7.208 (-4.605 to 19.022)	0.43	0.13
CD-RISC2	7.73 (1.57)	8.17 (1.50)	−0.438 (-1.105 to 0.229)	0.34	7.65 (1.48)	0.075 (-0.599 to 0.749)	0.99	0.16
Mastery Scale	17.64 (2.50)	17.44 (2.12)	0.198 (-0.772 to 1.169)	0.99	17.50 (1.88)	0.143 (-0.838 to 1.124)	0.99	0.87

*The bold values indicate statistical significance (p ≤ 0.05)*.

Similarly, the experience of victimization decreased significantly over 12 months (*F* = 4.009, *p* = 0.02) (Table 2) among participants, with the 12-month victimization scores significantly decreased compared to baseline (*p* = 0.05) (Table 2). There were no significant changes in anxiety or depression scores at 12 months, relative to baseline (Table 2). Similarly, there were no significant changes in substance use problem scores, resiliency scores, or PTSD symptoms at 12 months, relative to baseline (Table 2), and no significant improvements over the course of 12 months (Table 2).

An interaction analysis between time and baseline covariates (ACE scores, race, education level, having children, continuous work in the past year, place of birth, and immigration status) identified no significant association of any covariates with primary and secondary outcomes.

## Discussion

Although several studies have described the impact of interventions on homeless youth health and well-being, the literature on community-based interventions addressing GBV in this population is scant. Prior research with individuals experiencing GBV suggested that community-based, trauma-informed group interventions can improve mental health and quality of life by promoting personal growth and increasing self-esteem, empowerment, and coping skills in adolescent girls victims of sexual violence (23, 43, 44).

The study, part of a realist informed longitudinal evaluation of a community-based 16-week psychoeducation group intervention, identified improvements in quality of life, capturing physical, psychological health, social relationships, and the overall environment, and reductions in experiences of victimization among female identified youth experiencing GBV and homelessness 12 months following enrolment, with no significant changes in other health and well-being outcomes. Prior qualitative research by our team revealed that participants valued the safe, women-only space, the shared lived experiences, and tailored psychoeducation, suggesting the intervention is acceptable to service users (29). Furthermore, our findings of improved quality of life and reduced experiences of victimization are supported by prior qualitative findings by our team, highlighting perceived improvements in self-confidence, coping, health, interpersonal relationships, and future directedness as a result of program participation (29).

Our findings of improved overall quality of life scores are also consistent with prior research demonstrating positive impacts of some interventions on quality of life in female victims of GBV. Using a violence intervention program, Levas et al. (45) found increased scores in most domains of health-related quality of life after six weeks among youth aged between 8 and 18 years who attended a violence intervention summer camp in a Midwest urban city. Similarly, a study by Cripe et al. (46) found a trend toward improved quality of life among women survivors of intimate partner violence receiving supportive counseling focused on empowerment, compared to usual care.

Similar to other interventions such as community mobilization and group-based empowerment training (47), this trauma-informed psychoeducation intervention significantly reduced experiences of victimization 12 months after program entry. These findings are supported by prior research by Gilbert et al. (48), indicating that a brief intervention and referral to services may be effective in reducing physical and verbal intimate partner violence and physical violence in female survivors of GBV with substance use disorders. In contrast, several other intervention strategies such as awareness-raising campaigns (population-based prevention), home visitation and health worker outreach, personnel training, justice and law-enforcement interventions, have not been shown to reduce victimization in survivors of intimate partner violence or non-partner sexual violence (47). Among homeless youth, a mindfulness-based, cognitive, skill-building intervention for promoting risk detection to prevent various types of victimization suggested significant improvements in risk detection abilities in the intervention, compared to the control group (49).

We identified no significant improvements in substance use or trauma symptom severity, resiliency, or sense of mastery at 12 months. Lack of significant changes over time in these domains may suggest that the intervention was not effective in addressing these areas, and that more targeted interventions are needed to observe improvements in these outcomes. It is also possible, given the modest number of individuals with substance use disorders in our sample (32.8%), that the study was not sufficiently powered to identify changes in in this and other domains. In contrast, Gilbert et al. (48) found a significant positive impact on substance use over a 3-month period following a brief intervention and referral service for women survivors of GBV with substance use disorders. Similarly, among homeless youth, studies by Slesnick et al. (50, 51) demonstrated that a cognitive-behavioral intervention significantly improved measures of substance abuse among homeless intervention group participants, compared to control group participants. Given the association between female substance use and experiences of GBV in several studies (52–54), access to targeted interventions to address substance use are important program components for victims of GBV. Finally, studies have shown that social support and empowerment training can significantly reduce the symptoms of psychological distress such as anxiety and depression in victims of intimate partner violence (55, 56) and evidence-informed psychological interventions have the potential to reduce PTSD symptoms in women affected by GBV. Of note, PTSD, anxiety, depression and substance use were not specifically targeted in the intervention under study, although participants were offered information on relevant resources. To improve these outcomes, consideration should be given to engaging service user and provider stakeholders in program improvements, including seamless access to trauma-specific and substance use services, for those who need them.

Nearly 83% of study participants had an ACE score ≥ 4, much higher than the reported prevalence of ACE in a national sample of homeless youth with mental illness in Canada, where approximately half of the participants had an ACE score ≥ 4 (57). Several studies have identified a significant association between ACEs and different types of GBV such as intimate partner violence and family violence, and our findings add to the knowledge based on homeless youth specifically. We found no association between ACE scores and changes in outcomes over time.

GBV remains one of the most persisting and widespread problems faced by women and girls worldwide (58). Addressing the sequelae of GBV requires an understanding of the complex underlying social and/or economic factors, including housing stability. Given the high prevalence of GBV among girls and women experiencing homelessness (59), programs addressing GBV among homeless and marginally housed women and girls are urgently needed.

Trauma-informed care is a key component and philosophy in service delivery to survivors of GBV and has been found to play an essential role in the recovery of GBV survivors as well as homeless youth (60, 61). In addition to trauma-informed principles, trauma specific interventions may be needed to improve health outcomes of survivors of GBV experiencing homelessness, tailored to the needs of specific subpopulations. Although the literature on available interventions is growing, these is a paucity of studies examining how best to engage young women and girls experiencing homelessness in such interventions, which should be the focus of future research.

Study strengths include the uniqueness of the setting and paucity of prior research with this vulnerable population. Furthermore, the intervention was co-designed with service users, co-delivered with peer mentors, and found acceptable and valuable to the target population. Finally, the parent study included both qualitative and quantitative components, to comprehensively explore perspectives, experiences and outcomes of interest to all key stakeholders.

Although we estimated that our sample size had over 80% power to detect significant changes from baseline to 12 months in key outcomes, the small sample size was one of the limitations in this study. In addition, this is an observational, non-experimental study, and findings need to be interpreted with caution, although qualitative data support multiple perceived benefits of the intervention by study participants. Finally, the intervention was offered in Toronto, Canada, and findings may not be generalizable to other settings with different availability of resources and supports for this population. Further research should consider quasi-experimental studies with a control group of participants and randomized controlled trials, including wait-list controls.

## Conclusion

A brief, community-based, trauma-informed, group psychoeducation intervention may be helpful in improving the quality of life and reducing experiences of victimization among female identified youth experiencing homelessness and GBV. Further research, using experimental designs, is needed to examine the effect of interventions aiming to improve outcomes in this disadvantaged population, as well as to advance strategies to engage homeless youth in such interventions.

## Data Availability Statement

The raw data supporting the conclusions of this article will be made available by the authors, without undue reservation.

## Ethics Statement

The studies involving human participants were reviewed and approved by the Center for Addiction and Mental Health (CAMH) and the University of Toronto Institutional Research Ethics Boards. Written informed consent to participate in this study was provided by the participants' legal guardian/next of kin.

## Author Contributions

AB-F analyzed the data, and led the preparation of the manuscript. TR assisted in participant recruitment and data collection. WW assisted in statistical analysis. MM, AN, DK, and VS provided expert advice and aided in manuscript revisions. VS developed the study design and secured study funding. All authors contributed to the article and approved the submitted version.

## Conflict of Interest

The authors declare that the research was conducted in the absence of any commercial or financial relationships that could be construed as a potential conflict of interest.
